# Dietary Therapy for Von Gierke’s Disease: A Case Report

**DOI:** 10.7759/cureus.1548

**Published:** 2017-08-08

**Authors:** Mohammad Raza, Fehmina Arif, Pirthvi Raj Giyanwani, Saad Azizullah, Sonum Kumari

**Affiliations:** 1 Pediatric Department, Civil Hospital Karachi, Dow University of Health Sciences, Karachi, Pakistan; 2 Civil Hospital Karachi, Dow University of Health Sciences, Karachi, Pakistan; 3 Department of Oncology, Jinnah Post Graduate Medical Centre, Karachi, Pakistan

**Keywords:** glycogen storage disease, glycogen storage disease type i, von gierke’s disease, dietary therapy

## Abstract

Von Gierke’s disease, also known as glycogen storage disease (GSD) type 1A, is an autosomal recessive disease in which there is an inability to cleave glycogen to glucose because of a glucose 6 phosphate deficiency resulting in hypoglycemia and lactic acidosis. The patient may present with hepatomegaly and signs and symptoms of hypoglycemia. We diagnosed a case of Von Gierke’s disease in a seven-month-old female infant who was admitted for abdominal distension, vomiting, and lethargy for a duration of four months with characteristic rounded doll's face, fatty cheeks, protuberant abdomen, and massive hepatomegaly. Lab investigations showed low hemoglobin, low blood sugar level, lactic acidosis, hyperlipidemia, hyperuricemia, mild elevation of liver enzymes, and high anion gap metabolic acidosis. The diagnosis was confirmed with a liver biopsy and dietary treatment was started. This case report highlights the value of dietary therapy in improving the quality of life and survival and minimizing complications.

## Introduction

Von Gierke's disease, also known as glycogen storage disease (GSD) type l, is a rare autosomal recessive disorder of the metabolism in which there is an inability to break down glycogen into glucose due to the deficiency of enzyme glucose 6-phosphatase (G6Pase) [1]. Patients with GSD type 1 usually present at the age of three to six months with hepatomegaly and signs and symptoms of hypoglycemia; sometimes, they can present during the neonatal period with hypoglycemia and lactic acidosis [2]. Definitive diagnosis is confirmed by a liver biopsy and enzyme assay or by mutation analysis [3]. Appropriate dietary management decreases the metabolic abnormalities of the disease and its risk of chronic complications [4-5].

## Case presentation

A case being reported here is that of a seven-month-old female infant, a product of a consanguineous marriage, who was admitted for progressive abdominal distention, vomiting, and lethargy for four months. There was no history of seizure or recurrent infections. There was one admission in the past due to similar complaints. Her development was slightly delayed. There was a family history of the death of a cousin during infancy due to liver disease. Her own parents and elder siblings were healthy and alive.

**Figure 1:**
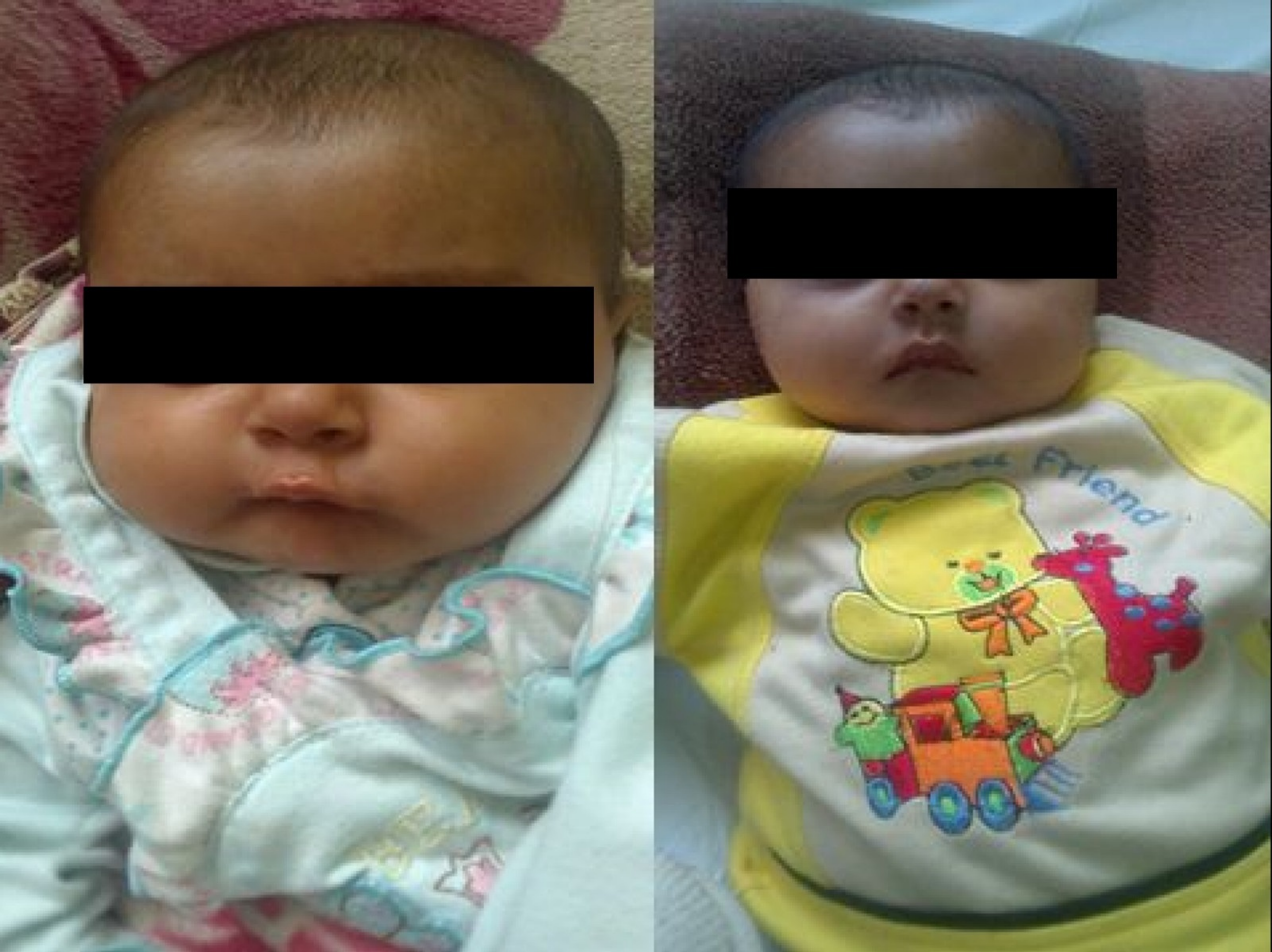
Image showing an infant having characteristic features of Von Gierke’s Disease, a rounded doll's face, fatty cheeks and protuberant abdomen

On physical examination, the lethargic infant had the characteristic features of Von Gierke’s disease: a rounded doll's face, fatty cheeks, and a protuberant abdomen (Figure 1); weight below the tenth percentile; and length below the fifth. Systemic examination was unremarkable except for a massive and firm hepatomegaly (liver span was 14 centimeter). The spleen and kidneys were not palpable.

When investigated, complete blood count (CBC) showed low hemoglobin, normal total leukocyte count, and differential leukocyte counts. The urine was negative for reducing substances. Apart from the low blood glucose level, blood chemistry revealed high lactic acid level, hyperlipidemia, hyperuricemia, mild elevation in liver enzymes, normal creatine kinase levels, and arterial blood gases revealed high anion gap metabolic acidosis. The diagnosis was confirmed by a liver biopsy. which demonstrated the mosaic pattern of enlarged hepatocytes, exhibiting abundant clear cytoplasm containing glycogen and lipid vacuoles.

Dietary therapy was started as frequent meals with high carbohydrate content, including nocturnal feeds. Later on, uncooked cornstarch, multivitamins, vitamin D, and calcium supplements were added. The patient's caretakers were advised to limit the intake of sucrose, fructose, and galactose-containing products. The patient’s general condition and hyperlipidemia improved on dietary treatment and she was advised regular follow-ups to monitor for long-term complications.

## Discussion

Von Gierke’s disease has an annual incidence of about 1/100,000 live births with signs and symptoms related to an inability to break down glycogen [4]. The age of presentation is variable. Carvalho et al. reported five adult patients with Von Gierke’s disease. Out of those five cases, four were diagnosed in the first six months of life, while the fifth one was diagnosed in adult life, after developing hepatocellular adenomas [4]. In a European study on GSD type I (ESGSD I), a majority of the patients presented between one and six months of age, very similar to that of our patient [6]. Howel RR et al. studied three siblings with classic glucose 6 phosphatase deficiency, who had a short stature with massive hepatomegaly, lactic acidosis, hyperlipidemia, and hyperuricemia. Similar findings were present in our patient [7]. The biochemical parameters of increased cholesterol, triglycerides, and uric acid have been reported in 76%, 100%, and 89% cases, respectively, as also present in our patient [8]. Without effective treatment, long-term complications occur, namely hepatic adenomas, renal dysfunction and nephrocalcinosis, osteoporosis, and gout [3].

## Conclusions

This case report highlights the value of early detection of the disease and strict adherence to dietary therapy, which leads to an improved quality of life, increases survival, and minimizes complications. Liver transplantation may reverse biochemical abnormalities.
